# BMI changes in children and adolescents attending a specialized childhood obesity center: a cohort study

**DOI:** 10.1186/1471-2431-13-216

**Published:** 2013-12-26

**Authors:** Albane BR Maggio, Catherine Saunders Gasser, Claudine Gal-Duding, Maurice Beghetti, Xavier E Martin, Nathalie J Farpour-Lambert, Catherine Chamay-Weber

**Affiliations:** 1 Pediatric sport medicine and obesity care program, Division of pediatric specialties, Department of Child and Adolescent, University Hospitals of Geneva and University of Geneva, 6, rue Willy-Donzé, 1211, Geneva 14, Switzerland; 2 Pediatric Cardiology Unit, Division of pediatric specialties, Department of Child and Adolescent, University Hospitals of Geneva and University of Geneva, Geneva, Switzerland

**Keywords:** Childhood obesity, Adolescents, Weight management, Behavioral techniques, Development

## Abstract

**Background:**

Multidisciplinary group therapies for obese children and adolescents are effective but difficult to implement. There is a crucial need to evaluate simpler management programs that target the obese child and his family. This study aimed to determine changes in body mass indexes (BMI) after individual family-based obesity intervention with a pediatrician in a specialized obesity center for child and adolescent.

**Methods:**

This cohort study included 283 patients (3.3 to 17.1 years, mean 10.7 ± 2.9) attending the Pediatric Obesity Care Program of the Geneva University Hospitals. Medical history and development of anthropometric were assessed in consultations. Pediatricians used an integrative approach that included cognitive behavioral techniques (psycho-education, behavioral awareness, behavioral changes by small objectives and stimulus control) and motivational interviewing. Forty five children were also addressed to a psychologist.

**Results:**

Mean follow-up duration was 11.4 ± 9.8 months. The decrease in BMI z-score (mean: -0.18 ± 0.40; p < .001) was significant for 49.5% of them. It was dependant of age, BMI at baseline (better in youngest and higher BMI) and the total number of visits (p = .025). Additional psychological intervention was associated with reduced BMI z-score in children aged 8 to 11 years (p = .048).

**Conclusions:**

Individual family obesity intervention induces a significant weight reduction in half of the children and adolescents, especially in the youngest and severely obese. This study emphasizes the need to encourage trained pediatricians to provide individual follow up to these children and their family. Our study also confirms the beneficial effect of a psychological intervention in selected cases.

## Background

The prevalence of childhood obesity is rising rapidly, resulting in increased prevalence of associated co-morbidities. About 20% Swiss children and adolescents are considered overweight and 5 to 8% of them are obese [1].

The most recent Cochrane review evaluated sixty-four randomized controlled trials in community setting of educational, behavioral and health promotion interventions for childhood obesity [2]. Authors concluded that comprehensive strategies involving the whole family to increase healthy diet and physical activity level coupled with psycho-social support and environmental change were more effective than those targeting the obese child alone. Another Cochrane review stated that “combined behavioral lifestyle interventions compared to standard care or self-help can produce a significant and clinically meaningful reduction in overweight in children and adolescents program” [3]. However, few studies analyzed the effectiveness of individual family intervention with trained pediatricians [4,5].

Therefore, the purpose of this project was to investigate changes in body mass index (BMI) in obese children and adolescents attending a specialized obesity care center in individual setting.

## Methods

### Study design and subjects

This was a cohort study including 283 patients (age 3.3 to 17.1 years, mean 10.7 ± 2.9) having at least two visits at the Pediatric Obesity Care Center of the Geneva University Hospitals between January 2008 and December 2010. Patients were followed for a minimum period of 6 months. Children were referred by their general practitioner, school nurses and families or by the Child and Adolescent Department of the Geneva University Hospitals.

We excluded children or adolescents if: 1) BMI z-score was normal, i.e. <1; 2) they attended the clinic only once; 3) they were registered in a structured multidisciplinary family-based behavioral group therapy during this period. Children with developmental delay or obesity related to genetic syndrome or endocrine disease were not excluded.

### Visits

All subjects visiting the center for the first time were attributed, according to their age, to a pediatrician trained in motivational interviewing and obesity care, assisted by a nurse trained in behavioral techniques. Adolescents above 14 years were seen by a pediatrician certified in adolescent medicine. The first consultation lasted an hour and follow-up visits lasted 30 to 45 minutes each. The type of treatment was defined at the first visit and was based on the “CONTREPOIDS©” protocol that we developed and described below. Some patients were referred to a psychologist when psychological problems such as depression or anxiety disorders were suspected. The intervals between each visit were defined according to the family needs and the clinic’s timetable (between 1 to 3 months). There was no intensive phase and the intervals between visits were kept constant.

The overall number of visits and BMI z-score between the first and last visit were calculated. If children were no longer attending the center at the date of final data collection (July 2011), we sent mails to their private medical practitioner, if known, in order to collect information about their current anthropometrics data (n = 137).

We also evaluated the current follow-up status and divided the children into three categories: 1) “current attendees” for those who were still attending the center in July 2011 (i.e. at least 6 months follow-up); 2) “improvement” for those who stopped the follow-up because of improved BMI z-score (with a reduction of at least 0.2); 3) “drop-out” for those who missed several scheduled contacts or stopped without notice.

### CONTREPOIDS©’ protocol

The CONTREPOIDS©’ obesity protocol has been developed according to the current evidence on obesity treatment [6,7]. This is an integrative approach including cognitive behavioral techniques (psycho-education, behavioral awareness, behaviors changes by small objectives and stimulus control) and motivational interviewing. All pediatricians and nurses working with this protocol have to follow a minimum of 3 days training to develop their ability in using these techniques. These skills were used to achieve goals in the domains of physical activity (active transports, sports, leisure time activities), sedentary behaviors (television viewing, electronic devices, etc.) food and drinking habits (food choice, portion size, hunger and satiety, beverages, etc.), as well as psychological issues and family support [8-11]. Assessing motivation and obstacles to behavioral change, and evaluation of modifiable lifestyle factors affecting body weight are the key points in the treatment. At the end of the consultation, one or two achievable lifestyle goals were chosen with the child/adolescent and his/her parents, depending of the age of the child. A handbook, with different thematic cards, was developed to help the caregivers in the treatment management of the patient and his/her family. A family therapist supervised the team in order to have a systemic view of the family and its difficulties when needed.

The treatment regiment was individualized and family-based, which means that parents were asked to be present at a part of the consultation. Families were encouraged to define their own goals with support and guidance from the pediatrician and nurse. Participation in physical activities was strongly supported. During follow up visits, individual objectives and difficulties encountered were discussed.

Informed, written consent was obtained from both parents and child during the prospective phase of the study. All subjects accepted to participate. The internal review board of the University Hospitals of Geneva approved the study.

### Measures

#### Medical history

At baseline, a detailed family and personal medical history was taken using a semi-structured interview. We recorded the age at weight gain (i.e. at what age the parents considered that their child’s weight began to cross the percentiles), and separated them into five categories for the analysis: 1) < 3 years, 2) 3 to 6 years, 3) 7 to 9 years, 4) 10 to 12 and 5) 13 to 16 years. The parent’s self-reported BMI were recorded. Parents were categorized into: both with normal weight; one overweight or obese; or both overweight or obese.

#### Anthropometrics

On each visit, we assessed body weight (kg) and height (cm). Body mass index (BMI) was calculated as weight/height squared (kg⋅m^-2^) and z-scores were derived using the World Health Organization references [12]. Overweight was defined as BMI between 1 and 1.99 SD and obesity above 2 SD.

#### Statistical analysis

Statistical analyses were performed using the SPSS software 18.0 (Chicago, IL). Data were normally distributed and presented as mean and standard deviation (SD). Statistical differences were analyzed using independent Student t-test or paired t-test and analysis of variance (ANOVA) with Bonferroni post-hoc test to compare the development per current attendance status and per age groups. We evaluated the correlations between variables using Spearman coefficient correlations. Differences were considered significant if P < 0.05.

## Results

### Patients’ characteristics at baseline

A total of 145 (51%) girls and 138 boys were included in the study. Mean BMI and BMI z-score were 26.4 ± 4.7 kg.m^-2^ and 2.7 ± 0.9, respectively. At baseline, 43 (15%) were overweight (mean age: 11.7 ± 2.6; 70% of girls) and 240 (85%) were obese (mean age: 10.5 ± 2.9; 48% of girls). Forty-five (16%) children were referred for psychological evaluation. Those with divorced/separated or widowed parents were more frequently referred (divorced: 23%, widowed: 20%, married: 10%; p = .003). Subjects were divided according to the age at the first visit: < 8 (18%), between 8 and 11 (46%) and > 12 years (36%).

### Development of patients

Mean follow-up time was 11.4 ± 9.8 months with a mean visit number of 4.6 ± 3.0. Twenty-nine percent of children (83) had two visits, 18% (50) had 3, 17% (49) had 4 and 36% (102) had more than 4 visits, respectively. In total, 151 (53%) subjects had 4 or more visits.

Mean BMI z-score was 2.7 ± 0.9 at baseline and 2.5 ± 0.8 at the end of the therapy, with a statistically significant mean overall BMI z-score change (-0.18 ± 0.40; range: -2.59 – 0.91; 95% confidence interval (CI): -0.23 to -0.14; p < .001) Boys had higher BMI z-scores at baseline than girls (2.9 ± 1.0 vs. 2.6 ± 0.7; p = .004). According to a change of BMI z-score of ±0.1, 49.5% of the patients (n = 140) decreased their BMI z-score, 36% remained stable (n = 102) and only 15% increased their BMI z-score (n = 42).

### Factors associated with the changes

Younger age, higher BMI at baseline (age: r = 0.195, BMI zs: r = -0.159; p < .001 for both) and number of visits (r = -0.133, p = .025) were associated with a better BMI z-score evolution. Gender, age at weight gain, parental marital or obesity status, intervals between visits, total follow-up time, current attendees status or psychological interventions were not correlated with BMI z-score changes.

Based on these results, we compared the development by attendee status and age groups. Results are detailed in Table 1. Children in all attendance status significantly improved their BMI z-scores. Interestingly, dropped out subjects were followed as long as non-dropped out. The number of children who had psychological intervention was similar between groups (p > .830).

**Table 1:** Development according to attendance status (n = 283)

	**Current attendees**	**Improvement**	**Drop-out**
Number, n (%)	104 (36.6)	21 (7.4)	158 (55.8)
Age (years)	10.1 ± 2.9^δ^	10.1 ± 3.1	10.7 ± 2.9^δ^
Gender girls, n (%)	60 (57.7)	10 (47.6)	75 (47.5)
Follow-up time (months)	14.6 ± 10.0^§^	11.1 ± 8.9	11.4 ± 9.8^§^
Number of visits	5.6 ± 3.1^§^	4.1 ± 2.4	4.6 ± 3.1^§^
BMI z-score at inclusion	2.8 ± 0.9	2.3 ± 0.7	2.7 ± 0.9
BMI z-score change	-0.22 ± 0.4^**^	-0.42 ± 0.6 ^δ,**^	-0.18 ± 0.4^δ,**^
Psychological intervention, n (%)	15 (14.4)	3 (14.3)	27 (17.1)

^δ^p < 0.05 between the 2 groups.

^§^p < .001 between the 2 groups.

*p < 0.05 and ** p < 0.01 for intra-group BMI z-score change.

**Figure 1:**
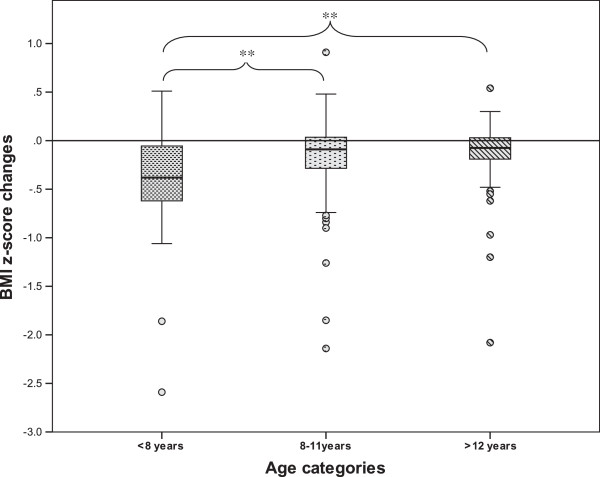
**BMI z-score development per age groups.** ** p < .001. Plain line represents no change in BMI z-score.

The development according to the age group is reported in Table 2. Children younger than 8 years had higher BMI z-score at baseline, were followed longer and had better outcome than other age categories (Figure 1). The outcome was not different between gender or adiposity categories (p > .05 for all age groups). The number of children who required a psychological intervention was similar between groups (p > .052), but the 8 to 11 years patients who had such therapy decreased their BMI z-score significantly more (-0.29 ± 0.62 vs. -0.12 ± 0.28; t = 2.05, p = .048) than the others.

**Table 2:** Development according to age groups (n = 283)

	**< 8 years**	**8 to 11 years**	**> 12 years**
Number, n (%)	51 (18)	131 (46)	101 (36)
Age (years)	6.3 ± 1.3	10.1 ± 1.2	13.7 ± 1.1
Gender girls, n (%)	27 (53)	74 (57)	44 (43)
Follow-up time (months)	13.2 ± 10.6^§^	12.5 ± 9.9^¥^	9.0 ± 8.8^§,¥^
Number of visits	5.1 ± 3.6	4.6 ± 3.0	4.3 ± 2.9
BMI z-score at inclusion	3.6 ± 1.2^§^	2.5 ± 0.7^§^	2.5 ± 0.7^§^
BMI z-score change	-0.40 ± 0.56^§,^*	-0.15 ± 0.37^§,^*	-0.12 ± 0.31^§,^*
Psychological intervention, n (%)	11 (21.6)	25 (19.1)	9 (8.8)

^§^p < .001 between the 2 groups.

^¥^p < .001 between the 2 groups.

* p < 0.001 for intra-group BMI z-score change.

Parental obesity appeared to be inversely correlated to BMI changes in the adolescent group only, with a decrease of the BMI z-score only when both parents had normal weight (t = -2.3, p = .027).

### Follow-up by their primary care physician

We received 63 (46%) responses to our mailing (137 messages sent). Eighteen (29%) children had not visited their primary care physician since their last visit at our center. For the 45 who visited their practitioner, only 13 (29%) had requested the appointment for their weight problem. In the remaining 32 children, this problem was tackled for 26 (81%) of them, and for 23 (72%) of them the practitioner had planned a next follow-up visit.

For these children, BMI z-score did not change between our last visit and the visit to the practitioner (mean BMI z-score change: 0.04 ± 0.5, p = .601). They had a mean of 3 consultations with their primary care physician.

## Discussion

The prevalence of childhood obesity is increasing worldwide and there is an urgent need to identify simple and effective interventions to treat a large number of patients with this condition. The primary aim of this project was to evaluate longitudinal changes in the degree of adiposity (BMI z-score) in children and adolescents attending an individual treatment program. Results of this study showed that about four medical consultations with trained pediatricians were effective to reduce the degree of adiposity in subjects. The BMI z-score reduction was of the same magnitude of more structured and intensive multidisciplinary group interventions. These positive changes were associated with lower age and higher BMI z-score at the first visit, as well as the number of visits.

Overweight children and adolescents from our cohort significantly decreased their BMI z-score by -0.18 ± 0.40, which is slightly better than the mean change of -0.15 kg.m^-2^ (95% CI: -0.21 to -0.09) reported in the last Cochrane review. Greater BMI z-score changes were observed in the youngest children, as previously reported by others [2,4]. In the last Cochrane review, the effects of intervention on BMI z-score by age subgroups were: 0 and 5 years: -0.26 kg.m^-2^; 6-12 years: -0.15 kg.m^-2^; and 13-18 years: -0.09 kg.m^-2^. These findings may be explained by the fact that young children are fully dependent of parent’s food and physical activity habits, as well as the home environment which can be more easily modified when parents are motivated. During adolescence, family support is also essential for weight management. However, parents have to struggle with their child’s development of autonomy and his growing ability to make decision and act on his own. Interestingly, BMI changes in adolescents were higher when both parents had normal weight, as previously suggested by Sabin and co-workers [4]. We may hypothesize that family support with a probably healthiest home environment and activities and/or favorable genetic predispositions may have contributed to the greater improvement in these adolescents.

The number of visits was also related to greater BMI z-score changes over time. In the 8 to 11 years group, the addition of a psychological consultation was also a factor influencing positively the outcome. Unfortunately too few adolescents (n = 9, 8.8%) could participate to such consultations due to the lack of personal resources in our center. Therefore, the sample size was probably too low to find an association in this age group. It is well known that overweight children and adolescents have a high rate of psychosocial co-morbidities such as anxiety, depressive mood disorder, adjustment disorder, or attention-deficit/hyperactivity disorder [13-16]. Depression seems to be especially frequent in this population, affecting almost 27% of them [17]. In fact, the risk of depression in obese adolescents is twice more frequent than in the general population of adolescents [17]. Screening for psychological co-morbidities at all ages is essential in order to treat them and to promote their adherence to recommendations and lifestyle changes [18,19].

During the follow-up, the drop-out rate was high with 56% of the children stopping the visits after 1 year, which is higher than the 21% reported in a comparable study [4]. However, follow-up time and BMI z-score changes were in the average of the cohort and of other studies. Various reasons could explain this high dropped out rate: satisfactory improvement, poor motivation or lack of results. Some families also appeared to be overwhelmed by psycho-social problems, with their child’s obesity being a secondary issue. Furthermore, the importance of personal, moral and sometime financial commitment in the treatment could be discouraging for these families. These data suggests the difficulties for families to engage in the long term and the challenge that pediatricians are facing in their practice.

The strength of this study was the large number of overweight or obese children and adolescents included in this longitudinal analysis. Compared to a group therapy, this outpatient therapy protocol was simple and could be easily performed by trained primary care providers in private practices or community care centers. Families appreciated the possibility of making an individual appointment according to their needs.

The main limitation of this study was the prospective longitudinal design instead of a randomized controlled trial. However, we do not think that it was ethical to leave overweight patients for a long period of time without treatment. Indeed, the majority of interventional studies have shown that without treatment, BMI z-scores increase [2]. Furthermore, several longitudinal studies in this population have demonstrated the same magnitude of BMI changes between uncontrolled and controlled studies. The second limitation was the non-standardized physical activity training: even if encouraged at least once a week, it was difficult to evaluate its impact on the results. The third limitation was the high drop-out rate that could weaken the efficacy of this approach. Nevertheless, we can observe that the results were good, even in the drop-out group, with a quite long follow-up time. It is also important to realize that not every child and family are ready to do some changes at the moment of the consultation. Some families were sent by their health care providers (physician, nurse) or family members with no self-motivation, and/or came to the consultation in order to find a quick and easy way to lose weight as proposed in many medias.

## Conclusion

This study highlights the fact that an individual and low-intensity family-based behavioral treatment during a year in an outpatient obesity clinic decrease BMI z-score in half of the children and adolescents, especially for the youngest and most severe obese children. The changes were of similar magnitude compared to intensive and complex multidisciplinary treatments previously described in the literature. Our findings also confirmed that a careful psychological evaluation is needed to enhance the success of the therapy, as many of them suffer from bullying, depression or other psychological condition that can interfere with the treatment. The greatest challenge is to promote and keep up motivation to limit drop-outs and sustain long-term behavioral changes. Further studies are required to evaluate the long-term results of individual therapeutic intervention.

## Abbreviations

BMI: Body mass index.

## Competing interests

The authors have no conflicts of interest to declare. This study was not supported financially and there is no non-financial competing interest.

## Authors’ contributions

AM: Dr. M conceptualized and designed the study, drafted and carried out the initial manuscript, and approved the final manuscript as submitted. CSG: Dr. S conceptualized and designed the study, reviewed and revised the manuscript, and approved the final manuscript as submitted. CG-D: Ms. G-D coordinated and supervised data collection, critically reviewed the manuscript, and approved the final manuscript as submitted. MB: Dr. B reviewed and revised the manuscript, and approved the final manuscript as submitted. XM: Mr. M coordinated and supervised data collection, critically reviewed the manuscript, and approved the final manuscript as submitted. NF-L: Dr. F-L reviewed and revised the manuscript, and approved the final manuscript as submitted. CC-W: Dr. C-W drafted and carried out the initial manuscript, and approved the final manuscript as submitted.

## Pre-publication history

The pre-publication history for this paper can be accessed here:

http://www.biomedcentral.com/1471-2431/13/216/prepub
